# Lifestyle Modification Experiences of African American Breast Cancer Survivors: A Needs Assessment

**DOI:** 10.2196/cancer.4892

**Published:** 2015-08-17

**Authors:** Selina A Smith, Mechelle D Claridy, Mary Smith Whitehead, Joyce Q Sheats, Wonsuk Yoo, Ernest A Alema-Mensah, Benjamin E-O Ansa, Steven S Coughlin

**Affiliations:** ^1^ Institute of Public & Preventive Health Georgia Regents University Department of Family Medicine, Medical College of Georgia Augusta, GA United States; ^2^ Department of Community Health and Preventive Medicine Graduate Education in Public Health Morehouse School of Medicine Atlanta, GA United States; ^3^ SISTAAH Talk Breast Cancer Support Group for Women of Color Miami, FL United States; ^4^ Institute of Public & Preventive Health Georgia Regents University Augusta, GA United States; ^5^ Institute of Public & Preventive Health College of Dental Medicine Georgia Regents University Augusta, GA United States; ^6^ Department of Community Health and Preventive Medicine Morehouse School of Medicine Atlanta, GA United States; ^7^ Division of Public Health Department of Health Science and Sustainability University of Massachusetts Lowell, MA United States

**Keywords:** African Americans, breast cancer, cancer survivors, nutrition, physical activity

## Abstract

**Background:**

Little is known about the rates of obesity among African American (AA) breast cancer survivors (BCSs), the availability and use of lifestyle modification methods suitable for this population, and the impact of changes in dietary intake and physical activity on health-related quality of life (HR-QoL).

**Objective:**

The objectives of the study were to describe obesity rates, dietary intake, and physical activity as lifestyle modification strategies; examine predictors of engagement in these strategies post diagnosis; and learn more about salient features of lifestyle interventions from AA BCSs participating in a breast cancer support group.

**Methods:**

The needs assessment included four components: (1) a literature review to determine existing lifestyle modification strategies of AA BCSs; (2) secondary data analysis of the 2010 National Health Interview Survey, Cancer Control Supplement to examine HR-QoL; (3) administration, to 200 AA BCSs, of an assessment tool relating to weight and breast cancer history, dietary intake, and physical activity through a variety of approaches (eg, Internet, mail, in-person, and telephone); and (4) focus group discussions to frame lifestyle interventions.

**Results:**

Preliminary findings indicate that AA BCSs are underrepresented in lifestyle intervention research, have disparities in HR-QoL outcomes, do not meet current cancer prevention guidelines, and have recommendations for effective strategies for lifestyle modification.

**Conclusions:**

As analyses of the needs assessment are completed, the research team is partnering with community coalitions and breast cancer support groups in Miami, Chicago, Houston, Los Angeles, and Philadelphia to develop community-engaged intervention approaches for promoting adherence to cancer prevention guidelines.

## Introduction

### Background

Based on Surveillance Epidemiology End Result data, an estimated 226,870 women were diagnosed with breast cancer (BrCa) in 2012, and 39,510 women died of the disease [1]. Nationwide, for most age groups, BrCa incidence rates are higher in Caucasian (white) women than in African-American (AA) women. Despite having a lower overall incidence, AA women have a higher incidence before 40 years of age and, at any age, are more likely to die from BrCa than other ethnic groups [2]. In addition to biological differences, this poorer outcome is attributed to late-stage diagnosis, unequal access to medical care, and lack of health insurance. Once BrCa is diagnosed, body composition also has a negative impact on clinical outcome; women who are obese at diagnosis have a 1.5- to 2.5-fold increased risk of recurrence and death compared to their normal weight counterparts [3-5]. Weight gain is common among BrCa patients after diagnosis and for those who become post menopausal after chemotherapy [6-11]. Effective long-term lifestyle modification is a target in reducing recurrence and enhancing prognosis among BrCa survivors (BCSs). Although a lifestyle change can halve the risk of recurrence and reduce the risk of BrCa-associated mortality by one third, many patients do not engage in lifestyle modification strategies (eg, changing dietary intake and enhancing physical activity). This is of particular concern among AA BCSs, as AA women have higher obesity rates than white women. Limiting use of these strategies among BCSs are psychosocial factors that may include anxiety and cancer-related fears [12,13], negative body image [14,15], depression [16-18], relationship changes [19], and/or financial stress [20].

Little is known about the rates of obesity among AA BCSs, the appropriate intervention methods available to them, and the utilization and impact of lifestyle modifications on health-related quality of life (HR-QoL). The goal of this assessment was to learn more about the lifestyle modification needs of AA BCSs.

### Objectives

The objectives of this study were to consider obesity rates, dietary intake, and physical activity as targets for lifestyle modification strategies; to examine predictors of engagement in these strategies post diagnosis; and to learn more about salient features of lifestyle interventions from AA BCSs. Since there are gaps in care for AA BCSs [21], a fragmented transition from active treatment to survivorship [22], and long-term implications of inadequate dietary intake on recurrence [23], Survivors Involving Supporters to Take Action in Advancing Health (SISTAAH) Talk, a BrCa support group, was selected as the study population because it is an untapped, indigenous resource for learning about and promoting lifestyle changes. The objective of SISTAAH Talk is to provide a forum for AA women to communicate about and make sense of their BrCa experience in order to achieve improved physical and mental health outcomes. We anticipate that inclusion of the target population in determining their lifestyle modification needs and experiences will result in development of testable interventions.

### Rationale for the Needs Assessment

Although incidence rates are 4% lower for AA women relative to white women, AA women are more often diagnosed with BrCa at younger ages and with more aggressive and advanced tumors [24,25]. Modifiable lifestyle risk factors related to energy balance [26] may contribute to racial/ethnic disparities in BrCa incidence and mortality.

Racial-ethnic disparities in modifiable BrCa risk factors are large and persistent, particularly between white and AA women [27]. Data from the Behavioral Risk Factor Surveillance System (BRFSS) relating to lifestyle factors revealed three disparity risk categories for AA women: (1) obesity (35.7% vs 23.7% for whites); (2) inadequate fruit and vegetable consumption (12.6% vs 17.4% for whites); and (3) physical inactivity (63.8% vs 50.3% for whites) [28]. Prevalence of overweight or obesity among AA women is 82% relative to 61% for white women [29]. Obesity and weight gain after BrCa diagnosis are associated with poorer outcomes, including decreased QoL, increased recurrence, BrCa deaths, and all-cause mortality [30]. For overweight and obese women, a sustained loss of 10% of initial weight reduces the risk of recurrence of a new primary BrCa [31]. According to the American Institute on Cancer Research (AICR), eating a healthy diet, maintaining a healthy weight, and being physically active can prevent about one-third of the most common cancers in the United States [32]. To reduce risk of recurrence, the AICR also recommends that cancer survivors adhere to cancer prevention guidelines.

## Methods

### Human Subjects

The Institutional Review Board at Morehouse School of Medicine approved the study protocol; participants received information on the study and consented participation.

### Literature Review

The first step in the needs assessment was completing a systematic review of the literature for English language articles in MEDLINE, MEDLINE In-Process, PubMed, and the Cochrane Library (Central Register of Controlled Trials). No date restrictions were applied, and free-text and Medical Subject Headings terms were used to identify studies including (but not limited to), lifestyle practices, dietary intake, physical activity, psychosocial factors, and QoL. Next, to search for areas of interest, terms were combined, for example, weight loss AND African American AND women AND interventions. The search did not include abstracts from conferences. Relevant, full-text publications that were potentially relevant were screened for inclusion based on the following criteria: (1) study design (prospective or retrospective observational studies, randomized clinical trials, or meta-analyses); (2) population (AA women); (3) lifestyle modification (diet, physical activity, weight control/loss); and (4) psychosocial factors (QoL, anxiety and cancer-related fears, negative body image, depression, relationship changes, financial stress).

### Secondary Data Analyses

Next, data from the national surveys were used to describe lifestyle and cancer risk behaviors of AA women. Baseline dietary intake, physical activity, and cancer risk behaviors of this population were established through an examination of the following secondary datasets.

The BRFSS is a state-based system of health surveys that collects information on health risk behaviors, preventive health practices, and health care access primarily related to chronic disease and injury [33]. The BRFSS measure of physical activity was used to capture typical weekly physical activity and scored in metabolic equivalent minutes/day (metabolic equivalent of task min-1 x day-1), including duration and intensity.

The National Health and Nutrition Examination Survey (NHANES) is a program of studies designed to assess the health and nutritional status of adults and children in the United States [34]. Our methods for measuring weight loss were adapted from the NHANES weight history questionnaire. By combining questions on self-directed diet changes (eg, “ate less food”, “ate less fat”, and “switched to foods with lower calories”) into a single item (“dieted on your own without joining a program or following a special diet book”) and separating commercial programs and self-help programs, modifications were made to the questions.

The National Health Interview Survey (NHIS) is the principal source of information on the health of the civilian, noninstitutionalized population of the United States [35]. The NHIS Cancer Control Supplement (CCS), administered every five years, focuses on issues pertaining to knowledge, attitudes, and practices in cancer-related health behaviors, screening, and risk assessment. The National Cancer Institute and the Centers for Disease Control and Prevention cosponsor the NHIS CCS. The cancer survivorship portion of the survey was included in our assessment tool and used to examine HR-QoL.

The final step was to condense lifestyle modification data. Results from the literature search and secondary data analysis were summarized to describe evidence-based, lifestyle modification efforts among AA women.

### Lifestyle Needs Assessment Tool Development

Validated scales (related to dietary intake/physical activity, weight loss history, and cancer risk) were selected from the datasets described above for inclusion in the needs assessment. Criteria for selection were based on the capacity of the measures to answer three questions: (1) Are AA BCSs aware of the relationship between lifestyle modification in preventing BrCa recurrence and enhancing QoL during and after treatment?; (2) If offered, will AA BCSs engage in lifestyle modification activities?; and (3) Which lifestyle modification strategies are most appealing to the targeted population?

The final tool assessed: (1) demographics (race/ethnicity, age, gender, education, income, religious affiliation, marital status, insurance, employment); (2) knowledge, attitudes, and beliefs (KABs) (BrCa survival/prognosis, diet/physical activity interventions); (3) BrCa history (BrCa diagnosis and treatment history, menopausal status, treatment side effects); (4) lifestyle modification needs and experiences (physical activity levels, dietary intake, self-reported current height and weight, self-reported weight at time of diagnosis, current weight loss attempt, number of weight loss attempts since BrCa diagnosis, weight loss methods tried); and (5) HR-QoL related to adjustment to BrCa, for example, cancer-specific HR-QoL (eg, emotional, physical, and social well-being), depression, fear of recurrence, diminished physical strength, change in relationships, change in body image, and financial stress.

Good readability, layout, and design were factors in developing the assessment tool. The Flesch reading ease (FRE) score was used to assess readability. The reading ease scores on the FRE scale are 0-100. If the score of a written text is less than 60, the document is considered difficult to read by the general public. To determine the time required to complete the assessment tool, 10 AA BCSs were interviewed. The time to completion was on average 45 minutes and unclear items were revised for clarity. The assessment tool was retested prior to finalization. The final needs assessment had a readability score of less than 80.

### Lifestyle Needs Assessment Tool Administration

A form letter to participants was developed. It stressed the usefulness of the information garnered from the tool to develop lifestyle interventions for AA BCSs and encouraged the subjects to complete the assessment tool.

Because the tool is an initial step in developing lifestyle interventions specific to the targeted population, engagement during the needs assessment phase is imperative to long-term success (eg, developing lifestyle interventions).

The final step was providing modes for administration of the assessment tool. Based on past research experiences with AA BCSs, we were aware of the need for multiple modes for administering the measure. In an assessment of BrCa gene-environmental interactions among multigenerational AA women with SISTAAH Talk members, we visited homes, met at infusion centers, communicated by telephone, and employed similar approaches to reach the targeted population. Assessment tools were self-administered electronically on the Internet, by email, or by mail to home addresses. They were also administered by an interviewer in-person or by telephone.

### Focus Group Discussions

There were four focus groups that were conducted with 8-12 BCSs engaged in 90-minute sessions to address intervention content. A moderator initiated each discussion with a structured set of questions. Sample size was determined based on the principle of saturation, which suggests that, with as few as four discussions, no additional information will be obtained. This qualitative sampling technique was used to ensure that perspectives across age groups were obtained. An interview guide was developed for this purpose. Responses were digitally recorded, transcribed verbatim, manually coded, and summarized. Qualitative content analysis was used to analyze the data [36]. Coding steps included developing preliminary themes, creating additional codes based on themes that arise, developing nonsubstantive codes, and producing detailed codes for analysis of specific topics. NVIVO 10, a computer-assisted qualitative data analysis software, was used to facilitate the coding process (ie, to determine the degree of agreement/disagreement across themes and to calculate interrater reliability scores) [37]. A process of double coding was used to overcome coder differences in reliability scores [38]. Recurring themes were identified, the research team came to consensus on coded themes, and themes were summarized for analysis.

### Statistical Analysis

Descriptive statistics were performed by determining means and SDs for continuous variables and frequencies and percentages for categorical variables from demographics, KABs, BrCa history, lifestyle modification experiences, and needs as well as psychosocial factors captured by the assessment tool. The t-score units, calculated by means and SDs for scores, were used to estimate the HR-QoL for four items and for physical and mental status. Multivariable linear regression analyses were performed to assess the influence of the multidimensional aspects of HR-QoL after adjusting for confounding demographic variables (age, marital status, and education) as covariates. The t-scores, odds ratios (OR), and related 95% confidence intervals (CI) were derived from multivariable analyses. The significance level was set at 0.05, and all tests were two-sided. All statistical analyses were accomplished with SAS version 9.2 (SAS Institute, Cary, NC).

## Results

### Findings From African-American Breast Cancer Survivor Studies

The literature review revealed that, although lifestyle changes can halve the risk of recurrence and reduce the risk of BrCa-associated mortality by one third, many patients do not engage in such strategies. Limited research on AAs exists because they have been underrepresented in studies examining health behaviors that improve BrCa survival [39]. The Women’s Healthy Eating and Living (WHEL) Study, one of the few studies with AA women, showed that, at baseline, AA survivors were more likely to be obese (45% vs 25% for whites), to consume more calories from fat (+3.2%), to have fewer servings of fruits (-0.7/day), and to be less successful at making and maintaining dietary changes than whites [40,41]. Greenlee et al [42] conducted a randomized controlled trial with the commercially available *Curves* program, following 42 Hispanic and AA BCSs for 6 months. The trial resulted in weight loss that was not maintained at 6 months after the intervention. A community-based pilot study of 24 AA BCSs who engaged in walking as physical activity [43], resulted in increases in steps walked per day and decreases in body mass index (BMI), body weight, and waist/hip circumferences, with most changes maintained at 3 months. A pre post design, that included one of two weekly sessions dedicated to exercise, was used to test a 6-month intervention with 23 AA BCSs [44]; participants experienced changes in weight, BMI, and social support. In a 16-week, home-based motivational exercise program for 13 AA BCSs, there was a post intervention increase in total minutes of physical activity and improved physical functioning [45,46].

### Secondary Data Analysis

In our secondary analysis of the NHIS 2010 data, female AA BCSs age 35 and older (n=62) were compared to AA female survivors of other cancers (SOCs) (n=74), and to AA women with no history of cancer (NHCs) (n=1566) of the same age. Differences in HR-QoL were assessed, including four items each and summary physical and mental health estimated in t-score units with one degree of freedom. All analyses were weighted and adjusted for age, marital status, and education. There were no statistically significant differences for BCSs and NHCs, but the SOCs reported poorer physical health relative to NHCs [t_1_=5.8, 95% CI 2.8-8.8]. Further, there were no statistically significant differences between BCSs and NHCs, but SOCs reported poorer mental health relative to NHCs [t_1_=3.3, 95% CI 0.6-5.9]. A comparison of differences between SOCs and NHCs showed three items in which SOCs were more likely to report poorer physical health relative to NHCs (ability to carry out physical activities, OR 3.4 95% CI 1.7-6.7, level of fatigue, OR 2.0 95% CI 1.1-3.7, and level of pain, OR 3.3 95% CI 1.3-3.9).

### Lifestyle Needs Assessment

AA BCSs were recruited from SISTAAH Talk, a BC support group in Miami, Florida (n=240; mean age 56.90 years; SD 11.80; range 25-92 years old), and they consented to complete a self-administered lifestyle assessment survey. More than half reported poor physical functioning; were overweight/obese (68%); did not limit portion sizes to control weight (89%); consumed <5 vegetables and fruits/day (75%), and >5 servings red (75%) and processed meats/week (94%).

### Focus Group Discussions

There were four focus group discussions (n=42; mean age 45.73 years; SD 7.91; range 35-75 years old) that identified barriers to and intervention approaches for enhancing dietary intake, and themes emerging from content analysis converged into the following categories: “talk” as central; peer-facilitated sessions; support group approach; no “pamphlet only” control group; “hands on” or interactive nutrition education; supporters (co-survivors); and community-based (not “placed”) research.

## Discussion

### Future Direction

With the successful implementation of this protocol for health needs assessment and the availability of preliminary findings from qualitative analyses, our study team is now planning health promotion trials in partnership with community coalitions and BrCa support groups in Miami, Chicago, Houston, Los Angeles, and Philadelphia to develop community-engaged intervention approaches for promoting adherence to cancer prevention guidelines. The focus of these proposed studies, which are at the protocol development and planning stage, is on increasing physical activity and improving diet among AA BCSs, with the goal of reducing risk of BrCa recurrence, improving survival, and increasing HR-QoL in this at-risk population. We anticipate that both feasibility trials and cluster-randomized controlled trials will be undertaken once the study protocols have undergone peer review and extramural funding is secured.

## Abbreviations

AA: African-American
AICR: American Institute on Cancer Research
BCSs: breast cancer survivors
BMI: body mass index
BrCa: breast cancer
BRFSS: Behavioral Risk Factor Surveillance System
CCS: Cancer Control Supplement
CI: confidence intervals
FRE: Flesch reading ease
HR-QoL: health-related quality of life
KABs: knowledge, attitudes, and beliefs
NHANES: National Health and Nutrition Examination Survey
NHCs: no history of cancer
NHIS: National Health Interview Survey
OR: odds ratio
SISTAAH: Survivors Involving Supporters to Take Action in Advancing Health
SOCs: survivors of other cancers
